# Enzyme-responsive hydrogel coating for *in situ* re-endothelialization of bioprosthetic heart valves

**DOI:** 10.7150/thno.131155

**Published:** 2026-04-08

**Authors:** Xuyue Liang, Qi Tong, Zhongwu Bei, Tianying Luo, Lin Ye, Meng Pan, Yun Yang, Bingyang Chu, Yongjun Qian, Zhiyong Qian

**Affiliations:** 1 Department of Biotherapy, Cancer Center and State Key Laboratory of Biotherapy, West China Hospital, Sichuan University, Chengdu 610041, China.; 2 Department of Cardiovascular Surgery, National Clinical Research Center for Geriatrics, West China Hospital, Sichuan University, Chengdu, 610041, China.; 3 Department of Ophthalmology, West China Hospital, Sichuan University, Chengdu, 610041, China.; 4 Research Laboratory of Plastic and Burns Surgery, West China Hospital, Sichuan University, Chengdu, 610041, China.

**Keywords:** hydrogel coating, bioprosthetic heart valves, antithrombotic, immunomodulation, re-endothelialization

## Abstract

**Background:**

Bioprosthetic heart valves (BHVs) replacement serves as a critical treatment for severe valvular heart disease. However, it often faces irreversible damage caused by thrombosis, inflammation, and calcification, severely limiting its therapeutic value for younger patients.

**Methods:**

Herein, we construct a biomimetic hydrogel coating loaded with cerium dioxide nanoparticles and vascular endothelial growth factor on the surface of BHVs material. This design aims to suppress material-induced thrombosis and inflammation, modulate the local microenvironment, achieve* in situ* re-endothelialization, and enhance the therapeutic potential of BHVs.

**Results:**

The findings demonstrate that the hydrogel coating improved the antithrombotic and anti-inflammatory properties of the BHVs material. Furthermore, i*n vivo* implantation in a rat abdominal aorta model exhibited effective *in situ* re-endothelialization.

**Conclusion:**

The hydrogel coating system developed in this study promoted *in situ* re-endothelialization of BHVs material and holds promise for extending its service lifespan. This work provides a strategy for enhancing the therapeutic potential of BHVs for younger patients.

## Introduction

As the third most prevalent cardiovascular disease worldwide, valvular heart diseases (VHDs) affect over 200 million individuals globally, with an estimated prevalence of approximately 2.5% and an annual mortality rate reaching 18%, posing a significant threat to human health and life 1. Currently, no available pharmacological interventions can cure VHD effectively, making prosthetic heart valve replacement the most viable treatment option for patients with severe VHDs 2. Worldwide, over 300,000 valve replacement procedures are performed annually, a number projected to reach 1 million by 2050, given the increasing prevalence of VHD 3, 4. Bioprosthetic heart valves (BHVs) have gained widespread clinical acceptance due to their superior hemodynamic performance and suitability for minimally invasive implantation 5, 6. However, with an average lifespan of approximately 10 years, which significantly limits the therapeutic value of BHVs, particularly for younger patients who may require subsequent open-heart surgeries for valve replacement 7, 8. There is an urgent need to explore construction methods for more durable transcatheter heart valves.

Native healthy valves are covered by a continuous endothelial-cell (EC) layer, which plays a pivotal role in maintaining hemostatic balance, regulating leukocyte behavior, and ensuring ionic homeostasis, thereby serving as a crucial foundation for long-term valve functional stability 9, 10. Consequently, promoting *in situ* re-endothelialization following BHVs implantation is essential for sustaining the valves' functional integrity and extending their service life.

Glutaraldehyde (Glut)-crosslinked decellularized porcine pericardiums (DPPs) are a crucial source for clinically available BHVs product. DPPs, as acellular extracellular matrix (ECM) materials, retain a relatively intact natural structure and provide an ideal microenvironment for the migration and proliferation of EC. While the immunogenicity generated by xeno-collagen and glycoproteins exposed during the DPPs decellularization process can be reduced by Glut crosslinking, this approach is not ideal in practical applications 11. Residual aldehyde group (rAGs), immunogenicity, cytotoxicity, and tissue injury induced during implantation can trigger thrombosis and inflammatory responses 12. The pathological microenvironment shaped by both thrombosis and inflammation not only disrupts the ECM structure but also inhibits EC adhesion and proliferation, thereby impeding re-endothelialization 13, 14. Therefore, effective control of thrombosis and inflammation is a prerequisite for achieving *in situ* re-endothelialization.

In our previous studies, we successfully introduced double bonds onto the surface of Glut-fixed BHVs material (Glut-PP) through covalent reaction between acrylamide-modified polylysine (pLys-MA) and rAGs 15, 16. On this basis, to further enhance the re-endothelialization potential of BHVs, a multifunctional hydrogel coating was constructed on the surface of the alkene-functionalized material using gelatin methacryloyl (GelMA) and [2-(methacryloyloxy) ethyl] dimethyl-(3-sulfopropyl) ammonium hydroxide (SBMA), and loaded it with cerium oxide nanoparticles (CeO_2_ NPs) and vascular endothelial growth factor (VEGF). Gelatin, a major component of ECM, possesses excellent biocompatibility and biodegradability, and its methacryloyl derivative has been widely applied in biomedical fields 17, 18. Concurrently, SBMA, a zwitterionic polymer renowned for its anticoagulant properties and its superior resistance to protein adsorption, is commonly used for surface modification of blood-contacting materials 19, 20.

The hydrogel coating composed of GelMA and SBMA is expected to inhibit immunogenicity, improve the biocompatibility of Glut-PP, and suppress platelet adhesion and activation, thereby achieving favorable antithrombotic properties. The favorable immunomodulatory ability of CeO₂ NPs has led to their broad application within the domain of biomaterials 21, 22. Encapsulation of CeO_2_ NPs within the hydrogel enabled their controlled release via enzyme-responsive degradation of GelMA in the inflammatory microenvironment. This mechanism is expected to improve the local immune milieu and create favorable conditions for EC adhesion. Additionally, given the pivotal role of VEGF in promoting EC proliferation, we co-encapsulated it within the hydrogel to synergistically enhance* in situ* re-endothelialization of the BHVs material 23.

**Scheme 1:**
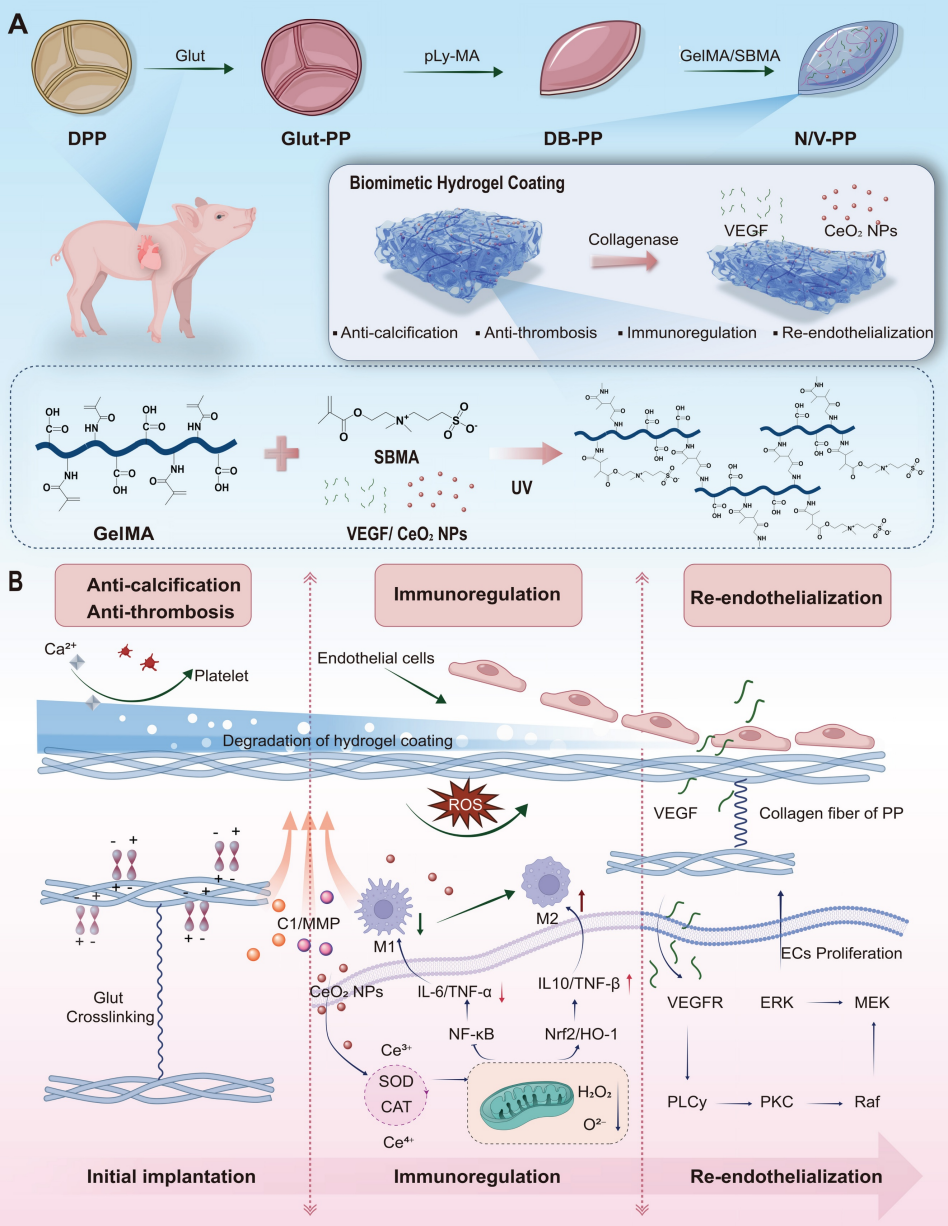
Design and working mechanism of a multifunctional hydrogel coating for BHVs. (A) Schematic of the stepwise fabrication of the hydrogel-coated material (N/V-PP). (B) Proposed temporal-sequential mechanism upon implantation: (i) The zwitterionic SBMA network confers immediate anti-fouling and antithrombotic properties. (ii) Inflammation-triggered, enzyme-mediated hydrogel degradation releases CeO₂ NPs that scavenge ROS and promote anti-inflammatory M2 macrophage polarization. (iii) Subsequently, sustained VEGF release facilitates endothelial cell recruitment and proliferation, achieving *in situ* re-endothelialization.

As shown in **Scheme 1**, the primary design intention is to first utilize the excellent biocompatibility and anti-adhesion properties of the coating to inhibit platelet adhesion and activation, thereby reducing thrombus formation. The hydrogel coating is progressively degraded by the elevated hydrolytic enzymes under ischemia-reperfusion and inflammatory conditions. The initially released CeO₂ NPs improve the inflammatory microenvironment through neutralizing reactive oxygen species (ROS) as well as shifting macrophage polarization toward the anti-inflammatory M2 phenotype, which aligns well with the requirements for early-stage valvular repair. Once the early inflammation is controlled, the focus of repair shifts to rapid endothelialization to prevent thrombosis and intimal hyperplasia. At this stage, the timely release of VEGF promotes endothelial cell proliferation, migration, and lumen formation, thereby advancing* in situ* re-endothelialization. To validate this hypothesis, we systematically evaluated the *in vitro* release profiles of CeO₂ NPs and VEGF, the interaction between the target material N/V-PP and macrophages, the antithrombotic properties of the material, its *in vivo* anti-inflammatory and anti-calcification effects, as well as its comprehensive performance within the circulatory system.

In summary, this study proposes a novel multifunctional coating strategy based on temporally coordinated release. Its core innovation lies in moving beyond the single-function approach typically pursued in existing BHVs surface modification schemes by mimicking the natural cascade of tissue repair through biomimetic design. Compared to common single-function coatings, one of the key advantages of this system is its temporally programmable functional synergy: rather than simply mixing antithrombotic (SBMA), antioxidant (CeO₂ NPs), and pro-endothelial (VEGF) components mechanically, we utilize an inflammatory microenvironment-responsive hydrogel as an intelligent carrier to achieve a precise temporal release logic of 'first antithrombotic/anti-inflammatory, then pro-endothelialization.' This ensures that during the peak inflammatory phase when endothelial cells cannot respond effectively, microenvironmental barriers are prioritized. Furthermore, the system possesses the capability for active microenvironment reprogramming: unlike coatings that merely provide passive anticoagulation or growth signals, our system, through the release of CeO₂ NPs, actively scavenges ROS and modulates macrophage polarization, reversing the implantation site from a "pro-inflammatory" to a "pro-repair" state. This creates an indispensable prerequisite for the subsequent effective action of VEGF. Therefore, the core contribution of this work lies in proposing and validating a new paradigm for valve material surface modification that is dynamic, intelligent, and functionally synergistic. Its design philosophy focuses on modulating the host response process itself, rather than merely applying a single effect, offering a new direction for developing next-generation long-lasting BHVs.

## Materials and Methods

### Materials

Gelatin were purchased from Aladdin, Glycidyl methacrylate (GMA), SBMA, Lithium phenyl-2,4,6-trimethylbenzoylphosphinate (LAP), 6-aminocaproic acid, cerium (III) nitrate hexahydrate, potassium persulfate, calcein-AM (Calcein-AM) /propidium iodide (PI), 6-aminohexanoic acid (EACA), 2,2'-azino-bis(3-ethylbenzothiazoline-6-sulfonic acid)-positive (ABTS^+^∙) titanium (IV) sulfate, lipopolysaccharide (LPS) and collagenase type I (C1) were obtained commercially from Macklin Biochemical Co., Ltd. CCK-8 and LDH Assay Kit was purchased from Beyotime Biotechnology Co., Ltd. (Shanghai, China). 4′,6-diamidino-2-phenylindole (DAPI) and tetramethylrhodamine isothiocyanate (TRITC)-phalloidin, matrix metalloproteinase-2 (MMP-2) and enzyme-linked immunosorbent assay (ELISA) kits with interleukin-6 (IL-6), VEGF, and interleukin-10 (IL-10) were acquired from Solarbio Science & Technology Co., Ltd. (Beijing, China).

### GelMA synthesis

GelMA was prepared according to established protocols 24. In brief, 10 g gelatin thrown into 100 mL deionized (DI) water in a three-necked, round-bottomed flask at 50°C with continuous stirring for 30 min. After the gelatin had completely dissolved, 6.4 g GMA was added dropwise to react under vigorous stirring at 50°C for 3 h. The crude product was centrifuged (13,000 rpm, 30 min) to remove aggregates, followed by dialysis (molecular-weight cutoff, 14 kDa) with DI water at 40°C for 6 days with daily water changes. The final product was freeze dried and characterized via hydrogen-1 nuclear magnetic resonance (^1^H NMR; 400 MHz, D_2_O, Bruker Daltonik GmbH, Bremen, Germany).

### Hydrogel fabrication and *in vitro* collagenase degradation test

Solutions of GelMA at different concentrations (5%, 10%, 15%, and 20%, w/v) and 3% SBMA were thoroughly mixed with 0.5% (w/v) LAP. The mixtures were poured into circular cell culture dishes and crosslinked under 365-nm ultraviolet (UV) light for 30 s. C1 solution (1 U/mL) was added to submerge the hydrogels. Samples were placed at 37°C under static conditions. At predetermined timepoints, the enzyme solution was aspirated, and the hydrogels were washed with phosphate-buffered saline (PBS) to terminate degradation. Degraded hydrogels were lyophilized and weighed to calculate mass loss percentage.

### Synthesis and characterization of CeO_2_ NPs

Synthesis followed modified protocols based on the literature 25. Briefly, 2.62 g EACA (20 mmol) was dissolved in 120 mL DI water and brought to 95°C under ambient conditions, followed by pH adjustment to 5.50 with 70 μL of concentrated hydrochloric acid (HCl, >35.0%). Next, 2.18 g cerium (III) nitrate hexahydrate (5 mmol) was dissolved in 100 mL DI water at 25°C. The cerium solution was added to the heated stabilizer solution, and the mixture was vigorously stirred (800 rpm), permitted to react for 1 min, and cooled to room temperature (RT). Particles were pelleted via centrifugation at 13,500 rpm for 30 min. The pellets were washed with acetone and recentrifuged under identical conditions. Purified CeO_2_ NPs were vacuum dried and redispersed in 10 mL DI water for further use. Hydrodynamic size and morphology were examined via transmission electron microscopy (TEM; JEM-2100). Crystal structure was determined via X-ray diffraction (XRD; D8 Advance; Bruker). Elemental composition and oxidation states were assessed via X-ray photoelectron spectroscopy (XPS; K-Alpha).

### Study on the antioxidant properties of CeO_2_ NPs

a) H_2_O_2_ scavenging test: Dispersions of CeO_2_ NPs (0.1, 0.2, 0.5, 1, 2, and 4 mg/mL; this concentration gradient was used thereafter) and various hydrogels were added to a 2 mM H_2_O_2_ solution, pure H_2_O_2_ solution as the control. The solution was incubated at 37°C for 5 min. The supernatant was collected and mixed with 200 μL of titanium(IV) sulfate solution (0.03 M) for 30 min 26. Measurements of absorbance (405 nm) were performed on a UV-Vis spectrophotometer (U-2900; Hitachi); b) ABTS^+^∙ radical scavenging capacity test: ABTS^+^∙ working solution was prepared by mixing an appropriate amount of ABTS^+^∙ reagent (7.4 mM) with an equal volume of potassium persulfate (K_2_S_2_O_8_; 2.6 mM). This mixture was kept in the dark to obtain the ABTS^+^∙ working solution. Subsequently, 0.2 ml working solution was added to NP solutions of different concentrations or ethanol (control), followed by a 6-min incubation. Measurements of absorbance (734 nm) were performed on a UV-Vis spectrophotometer; c) ·OH radical scavenging test: An equal volume mixture of salicylic acid (9 mM), ferrous sulfate (FeSO_4_; 9 mM), and hydrogen peroxide (H_2_O_2_; 8.8 mM) was prepared. Different concentrations of CeO_2_ NPs were added to the above mixture. A control solution without NPs was prepared and left untreated. All solutions were placed at 37°C for 30 min. Measurements of absorbance (510 nm) were performed on a UV-Vis spectrophotometer. The absorbance values for the control group and test groups were recorded as A_0_ and A_1_, respectively. The scavenging rates (H_2_O_2_, ABTS^+^∙ and ·OH radical) (%) were calculated as the following equation:

Scavenging rates (%) = [(A_0_ - A_1_) / A_0_] × 100%

### Preparation of Glut-PP

To prevent shrinkage during crosslinking, DPPs (5 cm × 5 cm) were fixed onto a support frame. The squares were then immersed in 0.625% (v/v) Glut solution and subjected to constant agitation at RT for 24 h. After processing, the frame-fixed Glut-PP was thoroughly rinsed with PBS to remove unreacted Glut and then immersed in a 2.5% (w/v) pLys-MA solution under constant agitation for 24 h, yielding double bond-functionalized PP (DB-PP).

### Preparation of hydrogel-coated DPPs

A solution was prepared by combining 10% GelMA (w/v), 3% SBMA (w/v), 0.5% LAP (w/v), VEGF (4 μg/mL), and CeO_2_ NPs (2 mg/mL) in DI water. Once this solution was thoroughly mixed, the lyophilized DB-PP was immersed in it. The coated material was then crosslinked under UV light (365 nm) for 30 s and labeled “N/V-PP.” The labels “V-PP,” “N-PP,” and “H-PP” denote materials loaded solely with VEGF, CeO_2_ NPs, and the hydrogel coating alone, respectively. The morphology of the resulting materials was observed via scanning electron microscopy (SEM; Nova Nano-SEM 450; Thermo Fisher Scientific), and the surface composition was examined via energy-dispersive X-ray spectroscopy (EDS).

### Study on release kinetics of CeO_2_ NPs and VEGF

Following UV cross-linking, hydrogels (1 mL) were immersed in PBS solutions either with or without C1 (1 U/mL)/MMP-2 (1 μg/mL). The solution was then incubated at 37°C. At predetermined timepoints, cumulative released amounts of CeO_2_ NPs and VEGF were subsequently determined using a UV-Vis spectrophotometer and a VEGF ELISA kit, respectively.

### Collagenase stability test of DPPs

Lyophilized samples (1 cm × 1 cm; n = 6) were weighed to obtain initial mass (M_0_). Samples were then incubated in 1 mL PBS containing C1 (125 U/mL) at 37°C for 24 h. After enzymatic treatment, the samples were rinsed with PBS, re-lyophilized, and reweighed to determine the final mass (M_1_). The mass loss rate (MLR) was calculated as follows:

MLR (%) = [(M_0_ - M_1_) / M_0_] × 100%

### Thermal-stability test

Lyophilized samples (Φ = 1 cm; n = 3) were hermetically sealed in aluminum crucibles. Shrinkage temperature was determined using a DSC 2920 instrument (TA Instruments, Newcastle, DE, USA).

### Water contact angle test

Lyophilized samples (Φ = 1 cm; n = 3) were mounted on glass slides with rough surfaces of DPPs adhered to substrates. Static water contact angles on smooth surfaces were measured using a contact angle measurement instrument (Biolin Scientific, Attension Theta, Sweden).

### Uniaxial tensile test

Samples (5 cm × 1 cm; n = 3) were equilibrated in PBS. Thickness was measured at three locations using a digital micrometer. Samples were gripped with an initial gauge length of 2.5 cm and stretched at 12 mm/min until fracture and calculated the relevant mechanical parameters based on the literature 27.

### Hemocompatibility test

Fresh rabbit blood containing sodium citrate as an anticoagulant was subjected to centrifugation (1500 rpm, 15 min), yielding two fractions: platelet-rich plasma (PRP) in the supernatant and erythrocyte concentrate in the pellet. Samples (Φ = 1 cm; n = 3) were seeded into a well of 48-well plate and underwent three washes with isotonic PBS, and mixed with 0.2 mL of 10× diluted erythrocytes plus 0.8 mL PBS. The negative and positive controls comprised 0.2 mL of diluted erythrocytes added to 0.8 mL of PBS or DI water, respectively. Following a 2-h incubation at 37°C, the samples were spun at 2500 rpm for 5 min. Measurements of absorbance (490 nm; A_1_) were performed on a Synergy H1 microplate reader (BioTek/Agilent). Negative and positive controls were denoted as A_N_ and A_P_, respectively. Calculation of the hemolysis ratio followed the equation:

Hemolysis (%) = (A_1_ - A_N_) / A_P_ × 100%

### Platelet adhesion and activation assay

Following addition to a 96-well plate, samples (Φ = 0.5 cm; n = 3) were subjected to a 1-h incubation with 100 μL PRP at 37°C. The samples underwent three additional rinses in PBS to remove non-adherent platelets. Platelet adhesion density was quantified using a LDH cytotoxicity detection kit (cytosolic-LDH release proportional to adherent-platelet count). Following fixation with 2.5% (w/v) Glut in 0.1 M PBS for 1 h, the samples were rinsed with PBS, dehydrated, and then observed under a SEM to evaluate platelet adhesion and activation.

### *In vitro* arteriovenous-shunt (*A-V* shunt) experiment

Animal experiments were approved by the Sichuan University Medical Ethics Committee (SUMEC; Accreditation No. 20250303092). Samples (1 × 1 cm^2^; n = 3) were immobilized on the luminal surface of sterile medical-grade silicone tubing. New Zealand white rabbit (2.5 ± 0.5 kg) was anesthetized with isoflurane. Systemic anticoagulation was achieved via heparin injection (305 U/kg). An extracorporeal arteriovenous circuit was established by anastomosing the proximal end of the cannula to the rabbits' common carotid artery and connecting its distal end to the contralateral external jugular vein. The configuration permitted circulating blood to maintain full contact with the test materials immobilized within the cannula lumen. After 2-h circulation, specimens were retrieved and perfused with PBS to remove non-adherent blood components. Macroscopic documentation was performed under standardized illumination. The specimens were treated with 2.5% (w/v) Glut in PBS for 1 h, followed by sequential dehydration through an ethanol gradient. Critical-point dried specimens were imaged via SEM.

### *In vitro* cytocompatibility evaluation

Samples (1 × 3 cm^2^; n = 6) were sterilized by UV irradiation followed by 75% ethanol immersion (24 h). After three rinses in PBS, samples were incubated in 5 mL Dulbecco's Modified Eagle's Medium at 37°C for 36 h to prepare material extracts. L929 mouse fibroblasts were plated in a 96-well plate (10,000 cells/well). Following a 12-h adhesion period, the medium was exchanged for 100 μL of material extracts. Following incubation for 24 or 72 h, the cells were washed thrice with PBS. After the addition of 100 μL CCK-8 reagent per well, and incubated for 2 h. Measurements of absorbance (450 nm) were performed. Cells cultured in medium without any test material were designated as positive control (set as the 100% cell viability reference). Absorbance values of all experimental groups (cells co-cultured with materials; A_1_) were compared with those of this positive-control group (A_P_), cell-free group was set as a negative control (A_N_) and the following formula was used to calculate the relative proliferation rate:

Relative proliferation rate = (A_1_ - A_N_) / (A_P_ - A_N_)) × 100%

### *In vitro* endothelial-cell adhesion and proliferation assay

Samples were cut into circular discs (Φ = 1 cm; n = 6), which were placed in a 48-well plate. The discs were sterilized using the method described above, followed by washing with PBS. Human umbilical vein endothelial cells (HUVECs) were plated at 2×10⁴ cells/well and cultured in a 5% CO₂ incubator for 1 or 3 days. After the culture period, the medium was exchanged for fresh medium containing 10% CCK-8 reagent After an additional 1-h incubation in a CO_2_ incubator, absorbance at 450 nm was measured. Additionally, the cells were stained with 3 μM calcein-AM PI for 10 min. Cell adhesion and viability were assessed and documented using an inverted fluorescence microscope (IX83, Olympis, Japan). Samples (Φ = 1 cm) were placed in confocal dishes. The discs were sterilized using the method described above, followed by washing with PBS to remove residual ethanol. HUVECs were plated onto the discs at 1×10⁴ cells/dish and cultured for 48 h. The cells were then subjected to fluorescent staining for cytoskeletal and nuclear visualization: they were fixed, permeabilized, and then stained with TRITC-phalloidin. Laser scanning confocal microscopy (Zeiss LSM 900) was subsequently employed for morphological observation and imaging of the cells.

### Macrophage activation and expression assay

Samples were punched into circular discs (Φ = 1 cm; n = 6) and placed in a 48-well plate. The discs were sterilized by UV irradiation and treatment with 75% ethanol, followed by washing with PBS to remove residual ethanol. RAW 264.7 cells were plated into 48-well plates (5×10³ cells/well). After 24-h co-culture, 1 mL LPS solution (10 μg/mL) was added to stimulate the RAW 264.7 cells for 2 h to induce activation. Following an additional 24-hour culture. The levels of IL-6 and IL-10 in the supernatants were measured using corresponding ELISA kits in strict accordance with manufacturers' instructions. In parallel, cells treated under the aforementioned conditions were plated on coverslips at 2×10^4^ cells per well to prepare cell monolayers. Following 24-h culture, dual-immunofluorescence (IF) staining was performed to detect CD86 and CD206 expression.

### Subcutaneous implantation in rats

This animal experiment was approved by SUMEC (Accreditation No. 20250303092). Sprague Dawley rats (male; 75 ± 5 g) were anesthetized. Following removal of fur from the dorsum, two 1-cm incisions were made. Samples (1 cm × 1 cm; n = 3) were implanted bilaterally into the subcutaneous pockets adjacent to the incisions. The wounds were subsequently sutured and disinfected with povidone-iodine. Following *in vivo* periods of 7 and 14 days, the samples were retrieved along with the surrounding native tissue. All tissues were fixed overnight in 4% (w/v) paraformaldehyde (PFA) solution, processed for paraffin embedding, and sectioned. Subsequently, the samples were processed for histological and immunohistochemical evaluation using H&E and IHC staining for macrophage polarization markers CD206 and inducible nitric oxide synthase (iNOS). The stained sections were imaged using a laser scanning confocal microscope (Zeiss LSM 880).

At 30 and 60 days post-implantation, additional samples were explanted along with surrounding tissues and fixed overnight in 4% (w/v) PFA solution. The samples alone were processed for paraffin embedding, sectioned, and stained with Alizarin Red to detect calcium deposition. Concurrently, for calcium quantification, the surrounding tissues were carefully dissected from the explanted samples, lyophilized, weighed, and then digested in 6 M HCl solution at 95°C for 2 h. The resulting solution was analyzed via inductively coupled plasma optical emission spectrometry (ICP-OES).

### Abdominal-aorta implantation in rats

These animal experiments were approved by SUMEC (Accreditation No. 20250303092). Samples (0.5 cm × 0.5 cm; n = 3) were rolled and sutured into a tubular shape with the smooth surface facing inward. Following anesthesia induction with 2% isoflurane, the abdominal aorta of female Sprague Dawley rats (200 ± 20 g) was surgically accessed. The above-mentioned tubular scaffold was implanted into the abdominal aorta via end-to-end anastomosis. Thirty days post-implantation, venous blood was collected for complete blood count and blood biochemical analysis, including aspartate aminotransferase (AST), alanine aminotransferase (ALT) and serum creatinine (sCr). Subsequently, the rats were euthanized, and major organs (heart, liver, spleen, lungs, and kidneys) and specimens were harvested. The specimens were fixed in 4% (w/v) PFA overnight. The inflammatory response, cellularization/tissue remodeling, and *in situ* endothelial regeneration were analyzed by immunostaining for CD86/CD163, vimentin (Vim), and CD31/ endothelial nitric oxide synthase (eNOS), respectively.

### Artificial intelligence (AI) tool usage declaration

DeepSeek was primarily used to check English grammar, diversify sentence structures, and enhance language fluency in the handwritten drafts of the "Introduction" and "Results and Discussion" sections. All AI-generated suggestions have been carefully evaluated, rewritten, and scientifically validated by the authors to ensure accurate representation of our original data and intent. All text processed with the AI tool underwent strict review, substantive editing, and final approval by the authors. AI suggestions were used as references; the original scientific facts, data interpretation, and logical reasoning were controlled, revised, and finalized by the authors.

### Statistical analysis

Data are presented as mean ± SEM and were analyzed by one-way ANOVA. Differences between means were determined using Fisher's least-significant-difference test.

## Results and Discussion

### Structure and anti-reactive oxygen species activity of CeO_2_ NPs

**Figure 1:**
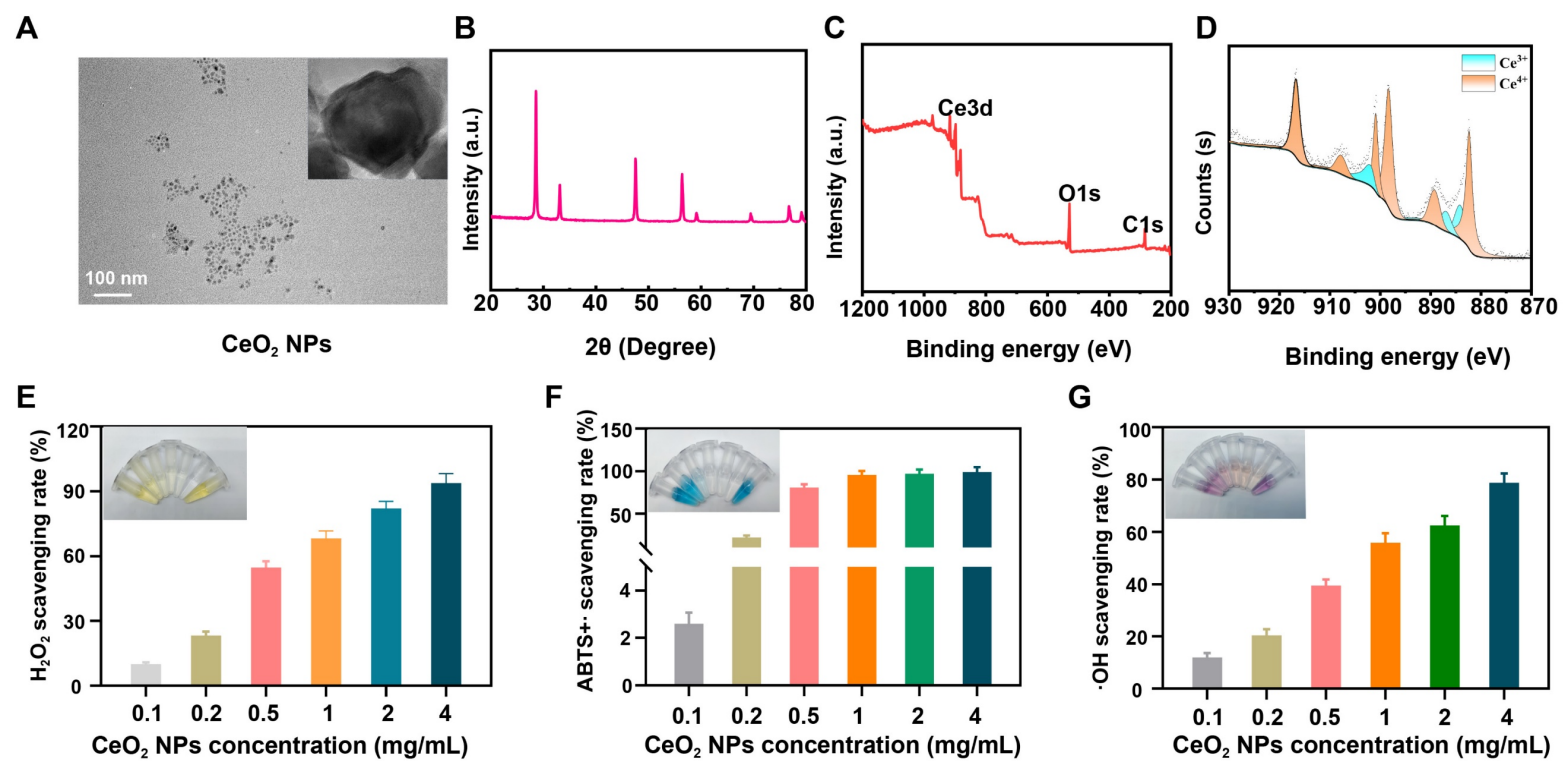
(A) TEM images of the CeO_2_ NPs. (B) XRD images of the CeO_2_ NPs. (C) Full spectrum and (D) Ce spectrum of CeO_2_ NPs. (E) H_2_O_2_, (F) ABTS^+^∙ and (G)·OH scavenging rate.

CeO_2_ NPs were successfully synthesized via a one-pot method and systematically characterized using TEM and XRD to determine their morphology and crystal structure. The results demonstrated that the synthesized CeO_2_ NPs exhibited uniform morphology and size distribution, with an average particle diameter of approximately 10 nm (**Figure 1A-B**). The XPS pattern (**Figure 1C**) confirmed a pure-phase cubic-fluorite structure, with all diffraction peaks matching the standard CeO_2_ crystal structure. In CeO_2_-based oxides, the coexistence of Ce^4+^ and relatively unstable Ce^3+^ forms a reversible-redox pair, endowing the material with exceptional catalytic activity. The reduction charge of cerium ions is compensated by a corresponding number of oxygen vacancies 28, a structural feature fundamental to the ROS scavenging capability of CeO_2_ NPs. XPS analysis further revealed the valence composition of cerium, with Ce^3+^ and Ce^4+^ accounting for 21.24% and 78.76%, respectively (**Figure 1D**), indicating their potential advantage in catalytically eliminating ROS and hydrogen peroxide 29.

Sustained inflammatory responses recruit and activate immune cells such as neutrophils and macrophages, leading to substantial ROS release. Elevated ROS levels can induce protein oxidation, deoxyribonucleic acid damage, and disruption of the ECM, thereby compromising cell membrane integrity and function. Further infiltration by inflammatory cells can prolong the inflammatory process, establishing a vicious cycle 30, 31. To systematically evaluate the antioxidant properties of CeO_2_ NPs, we assessed their scavenging capacities against H_2_O_2_, ABTS^+^∙ radicals and ∙OH radicals. When CeO_2_ NPs were mixed with H_2_O_2_ and visualized using titanium(IV) sulfate solution, the colorimetric signal decreased as NP concentration increased (**Figure 1E and S1A**), indicating rapid H_2_O_2_ decomposition. Total antioxidant capacity (TAC), as evaluated by the ABTS^+^∙ assay, showed a concentration-dependent fading of the characteristic bluish-green ABTS^+^∙ solution (**Figure 1F and S1B**), confirming significant radical-cation-scavenging activity. Moreover, in an Fe^2+^/H_2_O_2_ reaction system, CeO_2_ NPs markedly reduced ∙OH radical-induced signal intensity in a dose-dependent manner (**Figure 1G and S1C**), further demonstrating efficient ∙OH radical elimination.

In summary, the CeO_2_ NPs synthesized in this study exhibited excellent ROS-scavenging performance across multiple assays. They hold promise for mitigating ROS levels in the microenvironment that surrounds implantable materials and for suppressing inflammatory cascades, thereby providing a new material basis for enhancing the long-term implant stability of BHVs.

### Stability and mechanical properties of N/V-PP

**Figure 2:**
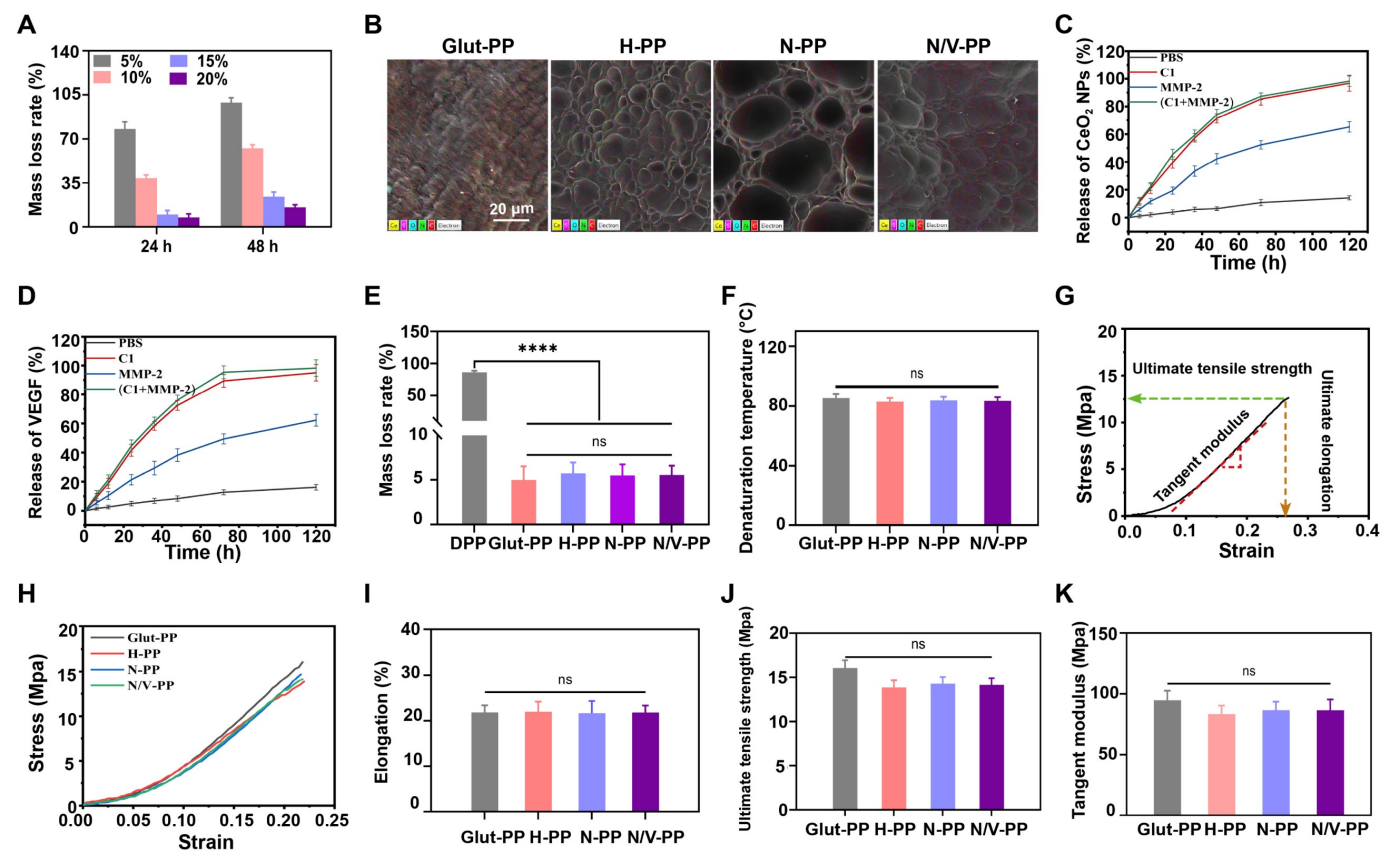
(A) MLR of hydrogels at different concentrations. (B) Element mapping images of DPPs. (C) Accumulative release rate of CeO_2_ NPs and (D) VEGF under in different enzymatic milieus. (E) MLR of DPPs (n = 6). (F) Thermal denaturation temperature of DPPs (n = 6). (G) Schematic diagram of mechanical testing parameters. (H) Stress-strain curve diagram of DPPs. (I) Elongation at break. (J) Ultimate tensile strength. (K) Tangent modulus (n = 3, ns means not significant, ****P < 0.0001).

Via ^1^H NMR spectroscopy, we confirmed successful synthesis of GelMA, with characteristic peaks in the chemical-shift range of 5.5-6.0 ppm indicating the successful grafting of methacryloyl groups onto the gelatin backbone (**Figure S2**). To identify the optimal hydrogel formulation, we systematically evaluated the degradation behaviors of hydrogels formed from different GelMA concentrations (5%, 10%, 15%, and 20%) in C1 solution (1 U/mL; **Figure 2A**). The results showed that the 5% GelMA hydrogel degraded almost completely within 24 h, a rapid degradation that could lead to burst release of encapsulated components. In contrast, hydrogels with concentrations ≥15% degraded slowly (mass loss < 30% after 48 h), which is unfavorable for sustained release of active agents. The 10% GelMA hydrogel demonstrated ideal degradation kinetics (approximately 60% mass loss after 48 h), enabling on-demand release of CeO_2_ NPs during the acute inflammatory phase for effective immunomodulation, and was therefore selected as the optimal concentration for subsequent experiments.

Next, pLys-MA was grafted onto the Glut-PP surface via a reaction between aldehyde and amino groups. Subsequently, we constructed a photo-crosslinked biomimetic hydrogel coating loaded with CeO_2_ NPs and VEGF on the BHVs surface. SEM images revealed the formation of a uniform porous hydrogel network on the DPPs surface, and elemental mapping further verified the successful incorporation of CeO_2_ NPs (**Figure 2B and S3**). ELISA results confirmed efficient encapsulation of VEGF within the coating (**Figure 2D**).

To assess the release behavior of the active components, we investigated the release kinetics of CeO_2_ NPs and VEGF in C1 and/or MMP-2 solutions. As shown in **Figure 2C-D**, the cumulative release of both components in the enzymatic solutions was significantly higher than that in the PBS control group, demonstrating the enzyme responsiveness of the hydrogel coating. This characteristic facilitates controlled release of the cargo in inflammatory regions *in vivo* (where enzyme concentrations are elevated), laying a foundation for subsequent immunomodulatory and pro-endothelialization functions.

BHVs must maintain long-term structural and functional stability after implantation. DPPs primarily consist of collagen-based ECM, which after implantation faces the risk of degradation not only by endogenous collagenases but also by various enzymes secreted by neutrophils during the inflammatory phase. Structural damage to the ECM can expose calcium-binding sites, accelerating valve calcification 12, 32. *In vitro* collagenase degradation assays and thermal-shrinkage tests indicated no significant differences in MLR or thermal-denaturation temperature among H-PP, N/V-PP, and N-PP compared with the Glut-PP control group (**Figure 2E-F**). Given that commercial BHVs universally employ Glut crosslinking, the modified BHVs developed in this study exhibited resistance to collagenase and thermal stability comparable to those of existing standard products. This stability primarily stemmed from the reaction between Glut and active amino groups on collagen, which effectively blocked enzymatic-cleavage sites while enhancing the compactness of the ECM structure through crosslinking.

Within the complex *in vivo* hemodynamic environment, BHVs must withstand continuous shear stress, cyclic flexure, and leaflet tension 33. However, unlike native valves, chemically processed BHVs are more susceptible to mechanical deterioration due to their altered ECM structure and lack of self-repair ability. Under long-term cyclic loading, irreversible damage gradually accumulates, ultimately compromising valve function. Because excellent mechanical properties are crucial to maintaining the long-term functional stability of BHVs, uniaxial tensile testing was performed to evaluate mechanical behavior (**Figure 2G**). The results revealed that compared with Glut-PP, the stress-strain curves of H-PP, N-PP, and N/V-PP showed a decreased slope (**Figure 2H**), indicating greater deformation under the same stress. This was potentially attributable to a toughening effect exerted by the hydrogel coating. Importantly, no statistically significant differences were observed between the modified groups and Glut-PP in terms of elongation at break, ultimate tensile strength, and tangent modulus (**Figure 2I-K**). Although Glut-PP has such limitations in its biocompatibility as thrombogenicity and inflammatory responses, its mechanical integrity has been validated by decades of clinical practice. These results indicate that our modification strategy effectively maintained the original mechanical properties of the material, thereby providing a stable structural foundation for its subsequent evaluations.

### Hemocompatibility and antithrombotic properties

As medical devices intended for direct, prolonged blood contact following implantation, BHVs must first exhibit excellent hemocompatibility. After they are implanted *in vivo*, inadequate biocompatibility can rapidly activate the coagulation system and induce thrombus formation. This process is initiated by the adsorption and activation of platelets on the material surface, which increases blood viscosity and triggers the intrinsic coagulation pathway, ultimately leading to thrombogenesis 34. The disruption of ECM caused by decellularization and the presence of residual Glut make BHVs prone to foreign-body reactions. These reactions subsequently recruit immune cells and activate platelets and thereby lead to leaflet thrombosis, which restricts leaflet mobility and causes stenosis and functional abnormalities, a common failure mechanism of BHVs.

**Figure 3:**
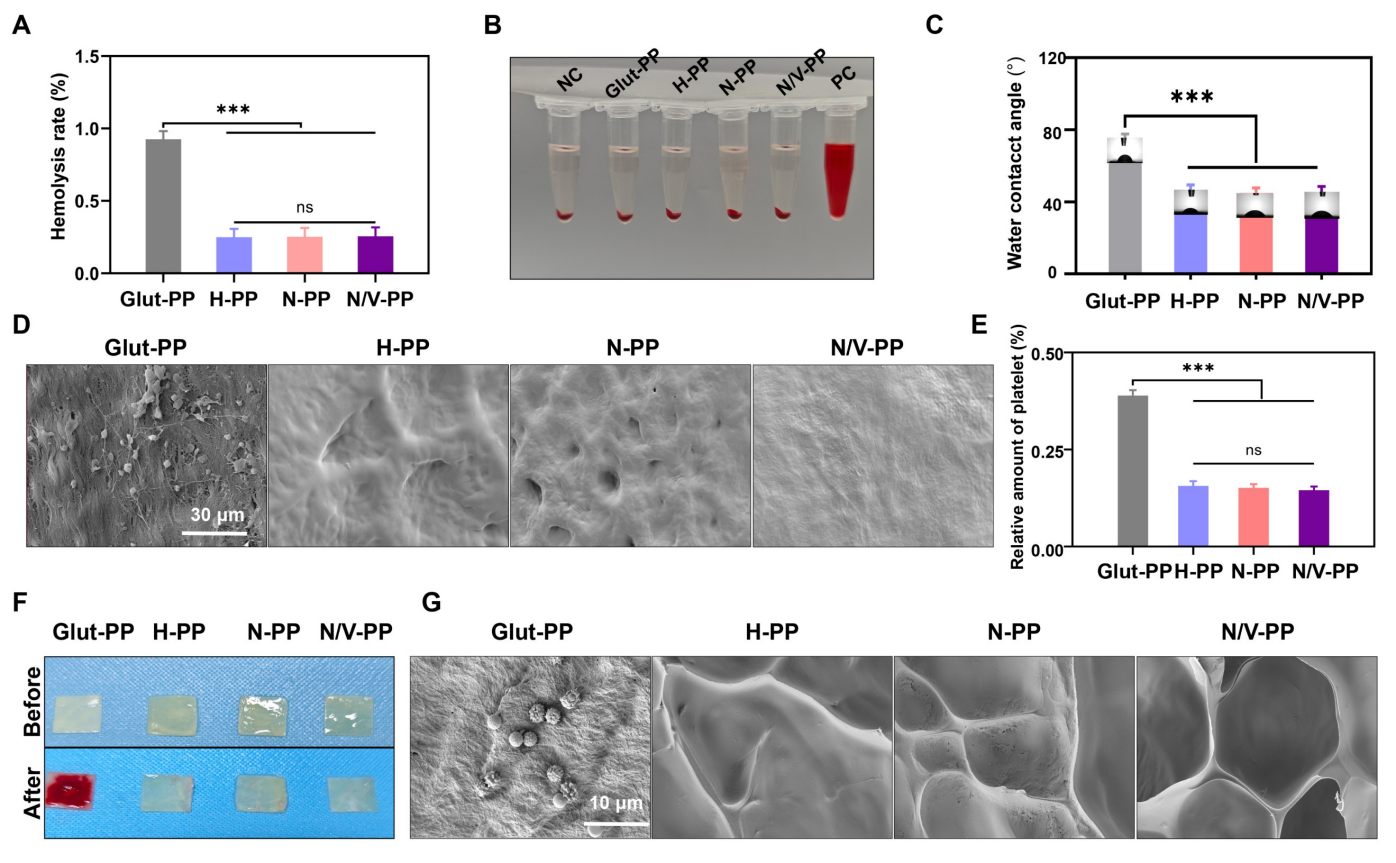
(A) Hemolysis rate of DPPs (n = 3). (B) Hemolysis images of DPPs. (C) Water contact angle of DPPs (n = 3). (D) SEM images of platelets adhered to DPPs surface. (E) Relative content of platelets adhered to DPPs (n = 3). (F) Blood adhesion on DPPs surface in *in vitro A-V* shunt model. (G) SEM images of blood components adhered to DPPs surfaces (ns means not significant, ***P < 0.001).

To evaluate the hemocompatibility of the materials, in this study, we initially performed *in vitro* hemolysis tests. The results (**Figure 3A-B**) demonstrated that hemolysis rates for all four sample groups were <1%, complying with relevant standards for blood-contacting medical devices and indicating negligible hemolytic activity and favorable hemocompatibility 35. Water contact angle measurements revealed that compared with Glut-PP, the H-PP, N-PP, and N/V-PP groups exhibited significantly reduced contact angles (**Figure 3C**), indicating markedly enhanced hydrophilicity, which helps minimize nonspecific binding to blood components. *In vitro* platelet adhesion assays further confirmed that the surfaces of H-PP, N-PP, and N/V-PP showed almost no platelet adhesion, whereas that of Glut-PP adsorbed a large number of platelets, with some showing signs of activation (**Figure 3D**). Results from the LDH assay kit were consistent with SEM observations, further validating these conclusions (**Figure 3E**).

To more directly evaluate the antithrombotic performance of the BHVs material, we conducted an *in vitro* A-V shunt experiment (**Figure S4**). After 2-h extracorporeal circulation, extensive thrombus formation was observed on the Glut-PP surface (**Figure 3F**), and SEM examination revealed substantial aggregation of blood cells (**Figure 3G**). In contrast, the surfaces of N-PP and N/V-PP remained largely unchanged from their pre-experimental states, with no significant blood cell attachment observed via microscopy. In summary, the biomimetic hydrogel coating effectively mitigated foreign-body response on BHVs surface, significantly suppressed platelet activation and thrombus formation, and thereby created a favorable microenvironment for EC adhesion and subsequent function.

### *In vitro* cytocompatibility and immunomodulation evaluation

Cytotoxicity induced by rAGs leads to cell death and suppresses cell adhesion and proliferation on BHVs, thereby impeding re-endothelialization; this is a key limitation to their long-term performance. Enhancing the surface cytocompatibility of the material is therefore essential for achieving rapid re-endothelialization. In this study, we formulated a biomimetic hydrogel coating to improve the material's cytocompatibility, used the immunomodulatory function of CeO_2_ NPs to ameliorate the inflammatory microenvironment, and incorporated VEGF to further promote EC proliferation.

**Figure 4:**
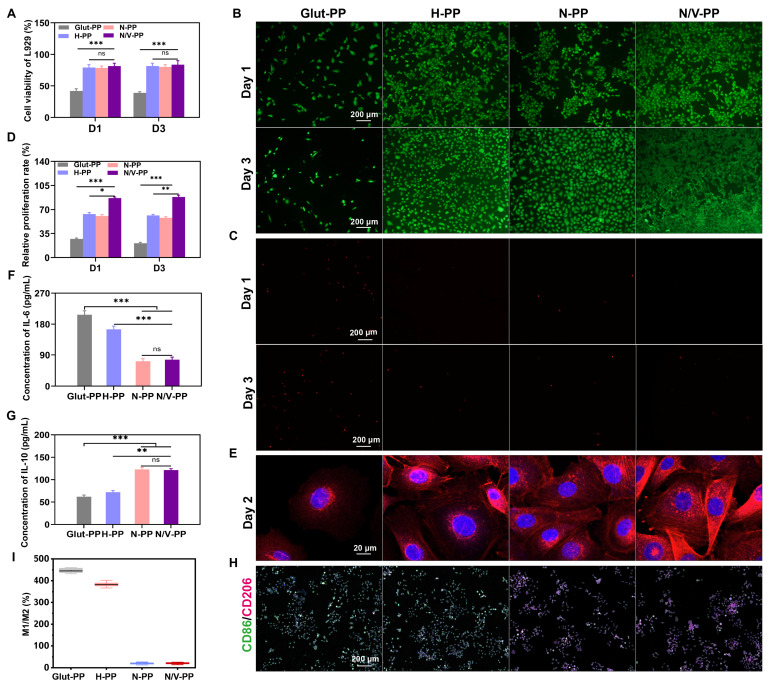
(A) Cell viability of L929 (n = 6). (B) Live/(C) Dead and (D) Relative proliferation rate of HUVECs after 1- and 3-day co-culture with DPPs (n = 6). (E) Morphology of HUVECs after 2-day co-culture with DPPs. (F) IL-6 and (G) IL-10 levels secreted by RAW264.7 after 2-day co-culture with DPPs (n = 6). (H) RAW264.7 cells IF staining images of CD86/CD206 and (I) The ratio of M1 to M2 polarized cells after 2-day co-culture with DPPs (n = 3, ns means not significant, *P < 0.05, ** P < 0.01 and *** P < 0.001).

First, we systematically evaluated the cytocompatibility of BHVs by co-culturing L929 cells with material extracts. CCK-8 assay results (**Figure 4A**) showed that viable-cell counts in the H-PP, N-PP, and N/V-PP groups were significantly higher than that in the Glut-PP group, indicating that the hydrogel coating effectively improved the material's cytocompatibility. To further investigate EC behavior on the material surface, we conducted co-culture experiments of HUVECs with DPPs to evaluate their adhesion and proliferation capabilities in order to provide an initial assessment of the material's re-endothelialization potential. After 1 and 3 days of culture, live/dead staining results (**Figure 4B-C**) revealed that the H-PP, N-PP, and N/V-PP groups supported higher live-cell counts and significantly fewer dead cells than the Glut-PP group. Semiquantitative CCK-8 analysis further confirmed that cell counts were significantly higher in the H-PP, N-PP, and N/V-PP groups than in the Glut-PP group, with the N/V-PP group also exhibiting markedly higher cell counts than the H-PP and N-PP groups (**Figure 4D**). Additionally, cytoskeletal morphology (**Figure 4E**) demonstrated that cells in the N/V-PP group exhibited more-extended and more-distinct actin structures, whereas those in the Glut-PP group had blurred boundaries and underdeveloped cytoskeletons. Taken together, these results indicated that the hydrogel coating effectively enhanced EC adhesion and viability on DPPs, while the release of VEGF further promoted cell proliferation. The coating thus preliminarily demonstrated favorable potential for promoting re-endothelialization.

Foreign-body reactions and implantation-associated inflammation can lead to infiltration and activation of immune cells such as macrophages. Activated macrophages polarize into one of two phenotypes: M1, in which macrophages secrete pro-inflammatory cytokines and generate ROS, exacerbating inflammation and leading to ECM degradation, valve thrombosis, and calcification; and M2, in which macrophages exert anti-inflammatory effects and contribute to tissue repair. Previous studies have reported that CeO_2_ NPs possess immunomodulatory functions and can promote macrophage polarization toward the M2 phenotype 36. To evaluate the immunomodulatory capacity of the BHVs material, we measured the expression levels of IL-6 and IL-10 in RAW 264.7 cells co-cultured with the materials. A shift toward a reduced IL-6 and an increased IL-10 secretory profile was evident in the N-PP and N/V-PP groups relative to the Glut-PP control; no significant changes were observed in the H-PP group (**Figure 4F-G**). To further directly assess the effect of the materials on macrophage polarization *in vitro*, we performed dual-IF staining for CD86 (an M1 marker) and CD206 (an M2 marker) of RAW 264.7 cells co-cultured with the materials for 2 days. As shown in** Figure 4H-I**, cells in the Glut-PP and H-PP groups were predominantly CD86^+^, indicating M1 dominance, whereas the N-PP and N/V-PP groups were mainly composed of CD206^+^ cells, suggesting M2 dominance. Collectively, these findings demonstrated that loading CeO_2_ NPs significantly enhanced the immunomodulatory function of the material, promoted macrophage polarization toward the M2 phenotype, helped alleviate inflammatory responses, and improved the local microenvironment, creating favorable conditions for EC adhesion and proliferation.

### Immunomodulation and anti-calcification testing in a rat subcutaneous-implant model

**Figure 5:**
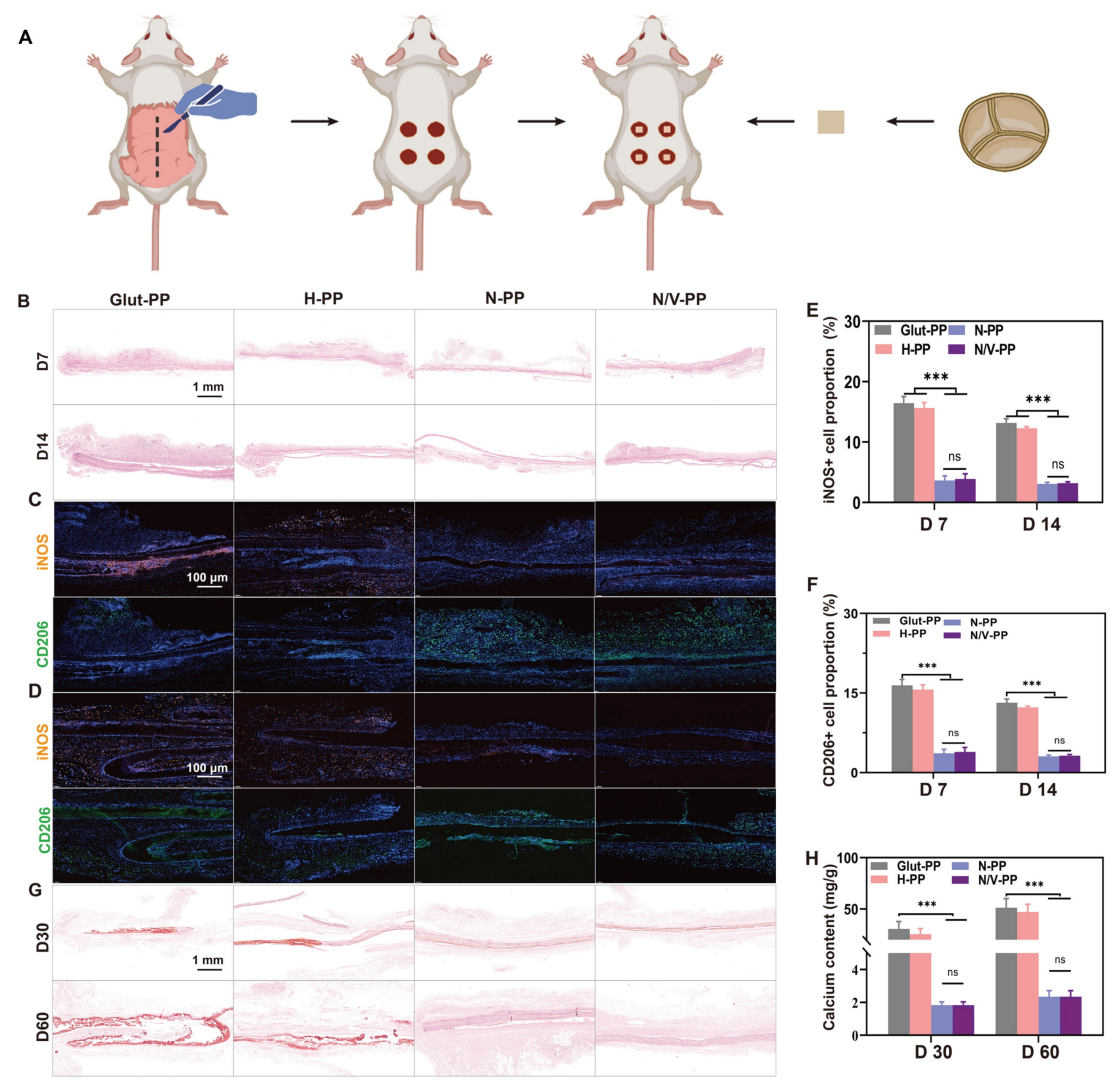
Performance of DPPs in rat subcutaneous implantation model. (A) Schematic illustration of subcutaneous implantation in rats. (B) H&E staining images. IF staining of iNOS and CD206 at (C) 7 and (D) 14 days after implantation. Relative fluorescence intensity of (E) iNOS and (F) CD206 (n = 3). (G) Alizarin red staining images. (H) Calcium ion content on DPPs (n = 3, ns means not significant, * P < 0.05 and *** P < 0.001).

The immunogenicity of BHVs and the tissue immune response elicited by implantation injury lead to the release of ROS, triggering oxidative stress (OS), which exacerbates inflammatory progression and induces tissue damage. CeO_2_ NPs were incorporated into the hydrogel coating owing to their favorable immunomodulatory capabilities 37. To investigate the *in vivo* immunomodulatory role of the hydrogel coating, we subcutaneously implanted differently treated DPPs and evaluated the ensuing inflammatory responses (**Figure 5A**). Histological analysis via H&E staining revealed notable differences in inflammatory reactions among the implantation groups. On day 7 post-implantation, substantial inflammatory-cell infiltration was observed in the tissues surrounding Glut-PP samples, with no significant mitigation by day 14. Similarly, the H-PP group exhibited no marked reduction in peri-implant inflammatory cells. In contrast, the N-PP and N/V-PP groups showed significantly diminished inflammatory-cell aggregation at both timepoints, indicating that CeO_2_ NPs effectively mitigated immune cell infiltration and foreign-body reactions (**Figure 5B**).

We further characterized the phenotypes of the inflammatory cells. INOS and CD206 were used as key markers for M1 and M2 macrophages, respectively. On days 7 and 14, mainly M1 macrophages surrounded the implants in the Glut-PP and H-PP groups, whereas implants in the N-PP and N/V-PP groups mainly exhibited M2 macrophages (**Figure 5C-F**). These results demonstrated that the hydrogel coating effectively modulated the immune response, suppressed inflammatory progression, and promoted its resolution, thereby improving the local microenvironment to facilitate EC migration and adhesion.

Calcification is generally considered a passive process, occurring independently of active regulation by recipient cells and driven predominantly by calcium phosphate deposition on cellular debris and fibrous components within BHVs. Calcification causes valve leaflet stenosis and hardening, leading to valvular dysfunction and blood regurgitation, which are major causes of BHVs failure. Although the underlying mechanisms are not fully clear, calcification is known to be closely associated with cytotoxicity, excessive inflammatory responses, and thrombus formation 38-40. We hypothesized that the biomimetic hydrogel coating could inhibit calcification by enhancing surface cytocompatibility, suppressing nonspecific-protein adsorption, and modulating immune responses. To evaluate its anti-calcification potential, samples were subcutaneously implanted in rats. Alizarin Red staining did not reveal significant numbers of calcified foci on the surfaces of N-PP and N/V-PP, whereas extensive red calcified areas were apparent on Glut-PP, and notable calcification deposits were also observed around H-PP (**Figure 5G**). These findings were corroborated by quantitative calcium ion analysis using ICP-OES, which showed significantly higher calcium content in the Glut-PP group than in the N-PP and N/V-PP groups (**Figure 5H**). We speculate that alleviation of the inflammatory response reduced inflammatory-cell infiltration, thereby decreasing the secretion of calcification-associated inflammatory factors and proteins and ultimately suppressing the occurrence of tissue calcification.

### *In vivo* abdominal-aorta implantation and long-term biosafety evaluation

**Figure 6:**
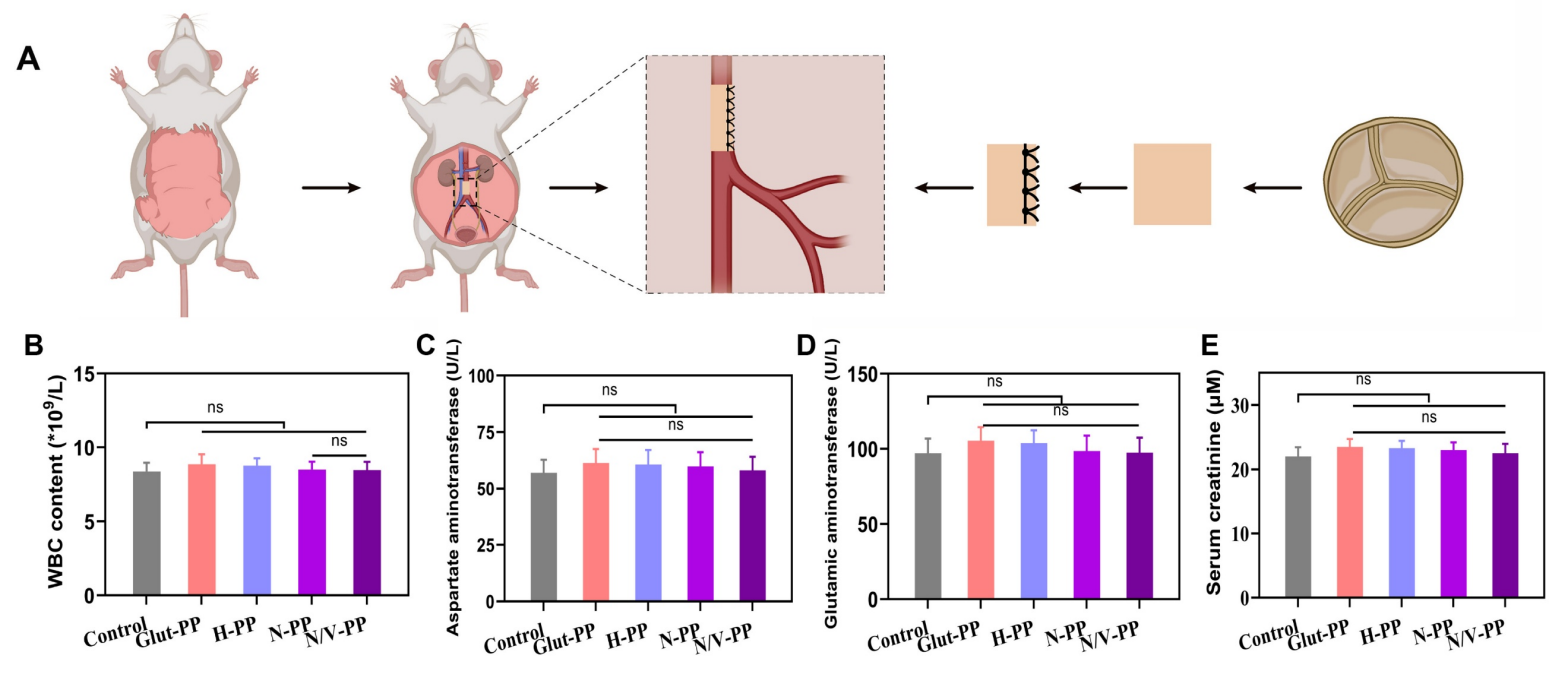
Biosafety assessment of the DPPs using a rat abdominal aorta model. (A) Schematic illustration of rat abdominal aorta implantation. (B) WBC content in peripheral blood (n = 3, ns means not significant). Serum levels of (C) AST, (D) ALT and (E) Scr.

Because BHVs are blood-contacting medical devices, their long-term biosafety is a core determinant of their clinical-translation potential. As mentioned previously, the hydrogel coating components GelMA and SBMA possess good biocompatibility; however, the biosafety of the incorporated CeO_2_ NPs under long-term *in vivo* conditions still requires systematic evaluation. Therefore, we established a rat abdominal-aorta implantation model to comprehensively assess the long-term biosafety of the material in multiple aspects (**Figure 6A**), including systemic inflammatory response, liver and kidney metabolic function, and the microstructures of major organs.

The presence of biotoxicity in NPs often triggers a systemic immune response in the host. Peripheral white blood cell count and neutrophil percentage serve as hallmarks of these inflammatory responses. Our experimental results (**Figure 6B and S5**) showed that, compared with Glut-PP group, the experimental groups loaded with CeO_2_ NPs (N-PP and N/V-PP) exhibited no statistically significant increase in these two parameters 1 month after implantation in rats. This finding strongly suggested that the release of CeO_2_ NPs from the material was minimal, or that even if a small amount was released, it did not initiate a significant inflammatory cascade in the body.

We primarily attribute this to two reasons. First, the hydrogel coating (GelMA/SBMA) acted as an effective physical barrier, firmly entrapping the CeO_2_ NPs and greatly restricting their burst release and systemic dissemination. Second, the inherent antioxidant-enzyme-mimicking activity of CeO_2_ NPs might have mitigated local OS induced by the implantation surgery itself, thereby preventing excessive activation of the inflammatory response. This observation aligns with the excellent anti-inflammatory capacity of the material noted in our previous experiment.

The liver and kidneys are major organs involved in the metabolism and clearance of NPs and are also the most susceptible targets of nanomaterial-induced toxicity. We assessed the core markers of liver and kidney function: AST, ALT, and sCr (**Figure 6C-E**). The data indicated no significant differences in these parameters between any group, biochemically demonstrating the absence of detectable hepatorenal damage within 1 month. This result was critical, since it ruled out the possibility of toxic effects from cerium ions—potentially released via biodegradation of CeO_2_ NPs—on hepatocytes and renal cells. We speculate that the sustained-release characteristics of the hydrogel coating, combined with the exceptional chemical stability of CeO_2_ NPs, collectively prevented rapid dissolution of the NPs and ion release, which is key to ensuring long-term biosafety.

To obtain more-direct pathological evidence, we performed H&E staining of major organs (**Figure S6A-E**). Observations revealed that the tissue structures of the heart, liver, spleen, lungs, and kidneys in rats from the experimental groups remained intact and clearly defined, with no signs of significant inflammatory-cell infiltration, tissue necrosis, fibrosis, or other pathological changes. These results morphologically corroborated the conclusions drawn from our hematological and biochemical analyses, indicating that the CeO_2_ NP-loaded coating did not induce local or distant organ damage *in vivo*. It is particularly noteworthy that no NPs accumulation or associated foreign-body reactions such as granuloma formation were detected in the spleen and kidneys, organs responsible for blood filtration, further implying a very low level of systemic exposure to the NPs.

Integrating the hematological, biochemical, and histological findings, we conclude that the CeO_2_ NP-loaded hydrogel coating exhibited excellent long-term biocompatibility after 1 month of implantation in the rat abdominal aorta without inducing significant systemic toxicity, immune reactions, or organ damage. This not only preliminarily verified the safety of this functionalized BHVs material but also laid a solid foundation for further *in vivo* functional evaluations.

### Functional evaluation of DPPs in a rat abdominal-aorta model

**Figure 7:**
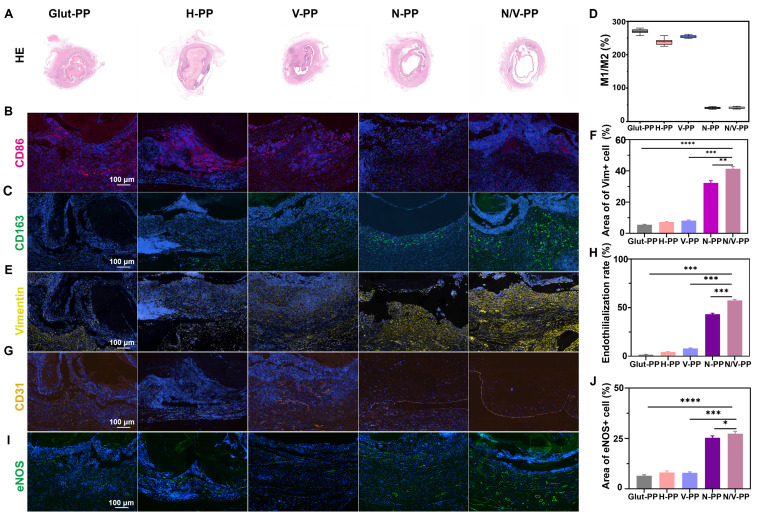
Performance of various DPPs implanted in the rat abdominal aorta for one month. (A) H&E staining of the explanted samples. IF images of (B) CD86 and (C) CD163. (D)The ratio of M1 to M2 macrophage content. (E) IF images of Vim. (F) Area of Vim+ cell. (G) IF images of CD31. (H) Endothelialization rate in different DPPs. (I) IF images of eNOS. (J) Area of eNOS+ cell (n = 3, ns means not significant, * P < 0.05, ** P < 0.01, *** P < 0.001 and **** P < 0.0001).

Building on previous findings, we further evaluated the biological performance of the hydrogel coating in a rat abdominal-aorta implantation model. Histological analysis 30 days post-implantation revealed that all material groups maintained vascular patency to varying degrees, with significant differences observed among them (**Figure 7A**). Compared with the Glut-PP group, the N-PP and N/V-PP groups exhibited larger luminal areas, demonstrating a significant lumen-preserving effect. The H-PP and V-PP groups showed intermediate results between the traditional group and the NP-treated groups. This phenomenon might be attributable to the fact that although the hydrogel coating initially maintained patency via its antithrombotic properties, inflammatory hyperplasia following hydrogel degradation could still lead to luminal stenosis as inflammation progresses. In contrast, the sustained release of CeO_2_ NPs, through effective immunomodulation, suppressed inflammation-related pathological hyperplasia, thereby providing crucial protection to maintain long-term luminal patency.

Analysis of macrophagic phenotypes further elucidated the regulatory effect of the materials on the local immune microenvironment (**Figure 7B-D**). Unlike the Glut-PP group, which was predominantly infiltrated by M1 macrophages, the N-PP and N/V-PP groups exhibited clear predominance of M2 macrophages. Notably, the H-PP and V-PP groups did not exhibit a similar shift in macrophagic phenotype. These findings indicated that while the base hydrogel material possessed good biocompatibility, its immunomodulatory capacity was limited. In contrast, CeO_2_ NPs actively directed macrophage polarization toward a reparative phenotype, converting the pro-inflammatory microenvironment into a pro-healing one. This mechanism provides a key explanation for the superior performance of the functionalized coating in tissue remodeling.

Achieving complete re-endothelialization is a central objective for extending the service life of BHVs. We systematically analyzed the effect of the functionalized coating on the re-endothelialization process via IF evaluation of multiple markers. Vim, a mesenchymal-cell marker, reflects the activities of tissue repair and matrix remodeling. CD31, directly indicates the extent of re-endothelialization. As shown in** Figure 7E-H**, our results demonstrated that the Glut-PP group exhibited minimal Vim+ cell infiltration and CD31+ EC coverage, indicating significant limitations in tissue remodeling and endothelial regeneration with traditional materials. In contrast, both the N-PP and N/V-PP groups demonstrated robust matrix remodeling and endothelial regeneration, with the N/V-PP group achieving a significantly higher endothelialization rate, 67.4%, than all other groups.

To further assess the functional status of the neointimal EC, we assessed eNOS expression. The eNOS is the key enzyme for nitric oxide production in EC, playing a central role in maintaining vasodilation and antithrombotic function. As shown in **Figure 7I-J**, the N-PP and N/V-PP groups exhibited strong positive signals for eNOS. The high expression level of eNOS in the N/V-PP group in particular indicated good functional activity of the new EC. Interestingly, the V-PP group, which received VEGF alone, did not significantly differ in eNOS expression from the Glut-PP group, despite the provision of pro-endothelial growth signals. This suggested that in an unmodified inflammatory microenvironment, mitogens alone are insufficient to achieve reconstruction of a functional endothelial layer. Conversely, CeO_2_ NPs created a favorable microenvironment, allowing VEGF to fully play its role in promoting functional endothelial regeneration.

Our findings showed that CeO_2_ NPs and VEGF could synergistically promote *in situ* re-endothelialization of BHVs material. This synergistic effect can be understood as a sequential therapeutic strategy of first improving the microenvironment and then promoting regeneration. CeO_2_ NPs, due to their antioxidative and immunomodulatory functions, scavenge excess ROS, suppress excessive inflammation, and polarize macrophages toward the pro-repair M2 phenotype, creating a microenvironment conducive to endothelial regeneration. Subsequently, VEGF acts on this optimized biological foundation to efficiently promote the migration, proliferation, and functional maturation of EC. More importantly, upregulation of eNOS expression indicated that this synergy not only facilitated the morphological reconstruction of the endothelial layer but also restored vascular function, which is crucial for maintaining long-term patency.

This study developed an enzyme-responsive multifunctional coating that promotes the *in situ* endothelial regeneration and integration of valves material through a meticulously designed cascade reaction. Its mechanism of action, as illustrated in **Scheme 1**, is as follows: upon implantation, the hydrogel coating prevents platelet adhesion and activation, thereby inhibiting thrombus formation. The elevated levels of C1 and MMPs in the local inflammatory microenvironment trigger the specific degradation of the hydrogel coating, promoting the preferential release of CeO₂ NPs. The reversible valence transition between Ce³⁺ and Ce⁴⁺ on the surface of CeO₂ NPs enables efficient scavenging of ROS 41-43. By clearing ROS, CeO₂ NPs can downregulate the activity of the NF-κB pathway, which is associated with a reduction in the production of M1-type cytokines such as TNF-α and IL-1β 44, 45. The clearance of ROS also relieves the inhibition on Nrf2, leading to the expression of antioxidant and anti-inflammatory genes such as heme oxygenase-1 (HO-1) 46, 47 . This, in turn, drives macrophage polarization toward the M2 phenotype, thereby shifting the lesion microenvironment from a pro-inflammatory to a pro-repair state. Within this improved microenvironment, the VEGF released from the coating not only directly stimulates the proliferation and migration of endothelial cells but also recruits endothelial progenitor cells to integrate into the nascent endothelial layer, accelerating the endothelialization process 48, 49. This "first anti-inflammatory and antithrombotic, then pro-endothelialization" temporally coordinated synergistic mechanism precisely mimics the ideal tissue repair pathway.

While the current study yielded encouraging results in the rat abdominal-aorta model, several limitations warrant consideration. First, the study focused on evaluating the biological effects of the multifunctional coating and did not directly quantify its interfacial bonding strength with the Glut-PP substrate. Although the coating's stability can be inferred from material chemistry principles and short-term *in vivo* observations, its long-term interfacial integrity under extreme conditions simulating the periodic high shear stress of heart valves remains to be rigorously verified through specialized mechanical testing (e.g., dynamic shear peel tests) in future work. The potential risk of coating delamination and its consequences (such as microembolism) represent critical safety issues that must be addressed prior to clinical translation.

In addition, native heart valves are subjected to high-intensity, multi-axial dynamic mechanical loads during the cardiac cycle. These primarily include high-magnitude, directionally periodic oscillatory shear stress generated by rapid blood flow, as well as cyclic tensile and compressive stresses caused by the frequent bending and extension of the valve leaflets. Consequently, the magnitude, frequency spectrum, and multi-stress coupling patterns of the forces in the rat abdominal aorta differ fundamentally from those in the valvular environment. This fundamental disparity in the mechanical environment may affect the comprehensive evaluation of our coating system's performance in the following aspects:

First, it concerns the long-term stability of the coating interface. The multi-axial cyclic stresses endured by the valve pose a severe challenge to the interfacial bonding strength of the materials. Coating adhesion that performs well under the relatively mild laminar shear of the aorta may face a higher risk of mechanical fatigue and delamination in the valvular environment—a risk not fully assessed in this model.

Second, it influences the kinetics of coating degradation and drug release. The enzyme-responsive nature of our coating design relies on the protease concentration in the local microenvironment. The complex flow patterns in the valvular region can significantly affect inflammatory cell aggregation and the spatial distribution of proteases. This may lead to deviations between the actual degradation rate of the coating and the release profiles of CeO₂ NPs/VEGF in the true valvular environment compared to the observations in the current model.

Finally, it relates to the functional maturation and long-term fate of the neointimal layer. It remains to be verified in future, more physiologically relevant mechanical models whether the confluent endothelial layer formed under the laminar flow conditions of this model can maintain its antithrombotic, anti-proliferative homeostatic functions under the intense oscillatory shear stress characteristic of valves.

Therefore, the excellent pro-endothelialization results observed in this study must be interpreted within the context of its specific experimental conditions: they primarily reflect the exceptional bioactivity and inherent potential of this multifunctional coating to promote endothelial repair under relatively ideal, low-shear stress conditions. These findings constitute a crucial proof-of-concept, providing a solid theoretical foundation for subsequent research. The core of future work must inevitably build upon this basis, utilizing *in vitro* biomimetic systems or large animal orthotopic heart valve implantation models that can simulate the true mechanical environment of valves to validate the coating's long-term durability, adaptability, and functionality, thereby advancing its clinical translation.

## Conclusion

In this study, we constructed a biomimetic hydrogel coating to enhance the comprehensive performance of BHVs. N/V-PP maintained the favorable mechanical stability inherent to BHVs material. Furthermore, the coating, composed of biomimetic materials with excellent biocompatibility and zwitterionic components possessing strong antifouling efficacy, effectively reduced thrombus formation. The stimuli-responsive release of anti-inflammatory CeO_2_ nanoparticles enabled immunomodulatory functions and provided a conducive microenvironment for endothelial-cell adhesion. The release of vascular endothelial growth factor promoted EC proliferation, facilitating the achievement of *in situ* re-endothelialization. The superior material properties of N/V-PP, combined with its antithrombotic activity, immunomodulatory capacity, and ability to support *in situ* re-endothelialization, position it as a promising candidate material for next-generation BHVs. This approach holds considerable promise for enhancing the service longevity of BHVs product.

## Supplementary Material

Supplementary figures.

## Data Availability

Any additional information required to reanalyze the data reported in this work paper is available from the lead contact upon request.
