# Supplementary material for: Enzyme-responsive hydrogel coating for in situ re-endothelialization of bioprosthetic heart valves
Source: Theranostics. 2026 Apr 8;16(11):6032–50. doi: 10.7150/thno.131155 (PMC13142126; doi:10.7150/thno.131155)
Supplement: Supplementary file 1 — Supplementary figures. [file thnov16p6032s1.pdf]

Supplementary files

Enzyme-responsive hydrogel coating for *in situ* re-endothelialization of bioprosthetic heart valves

Xuyue Liang[1], Qi Tong[2], Zhongwu Bei[1], Tianying Luo[1], Lin Ye[3], Meng Pan[1], Yun Yang[4], Bingyang Chu[1], Yongjun Qian[2]*, Zhiyong Qian[1]*.

1. Department of Biotherapy, Cancer Center and State Key Laboratory of Biotherapy, West China Hospital, Sichuan University, Chengdu 610041, China.
2. Department of Cardiovascular Surgery, National Clinical Research Center for Geriatrics, West China Hospital, Sichuan University, Chengdu, 610041, China
3. Department of Ophthalmology, West China Hospital, Sichuan University, Chengdu, 610041, China
4. Research Laboratory of Plastic and Burns Surgery, West China Hospital, Sichuan University, Chengdu, 610041, China.

*Corresponding authors: E-mail: anderson-qian@163.com, qianyongjun@scu.edu.cn.

Results

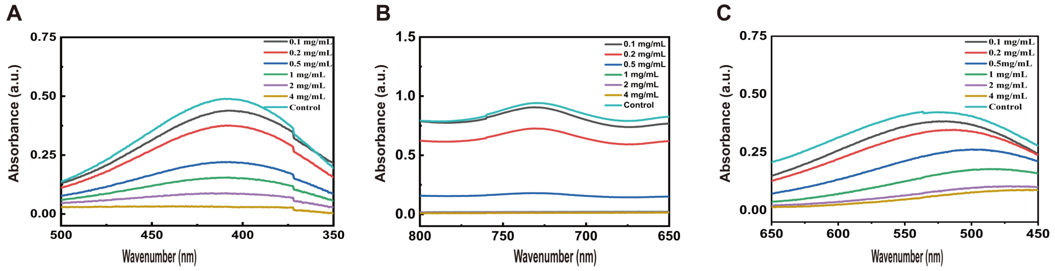

Figure S1 UV-Vis spectroscopy of (A) H_2O_2 hydrogen peroxide (B) $ABTS^{+\cdot}$ and (C) ·OH treated with different concentrations of CeO_2 NPs.

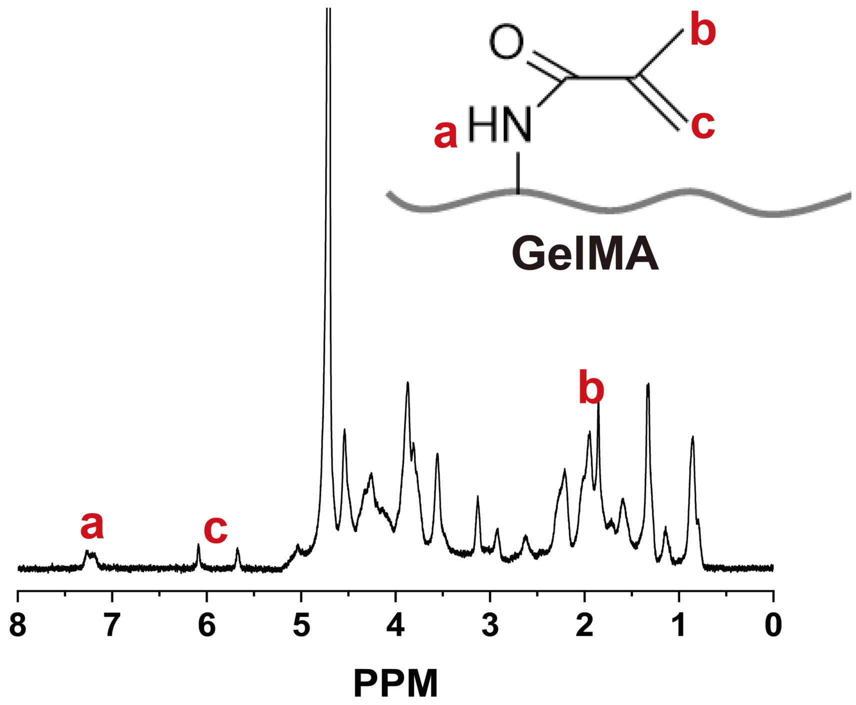

Figure S2 ^{1}H NMR spectrum of GelMA.

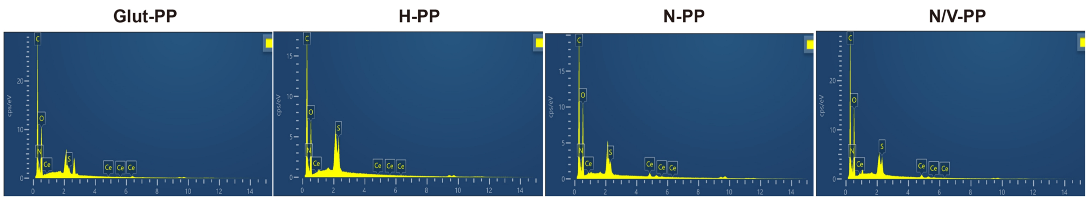

Figure S3 EDS spectrum of DPPs.

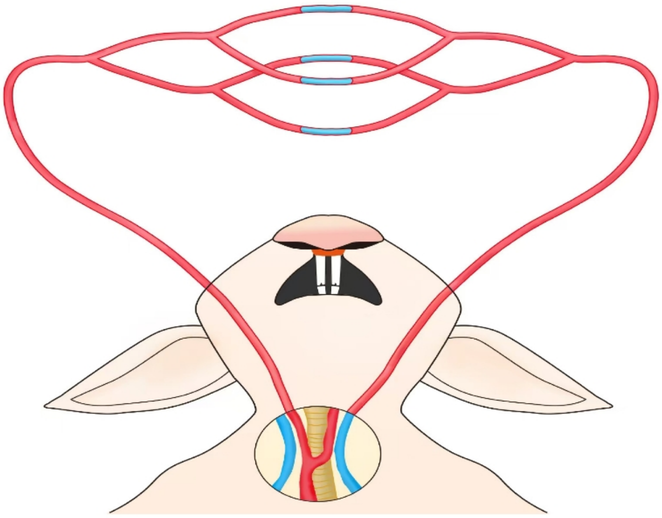

Figure S4 Schematic diagram of rabbit *in vitro* A-V shunt experiment.

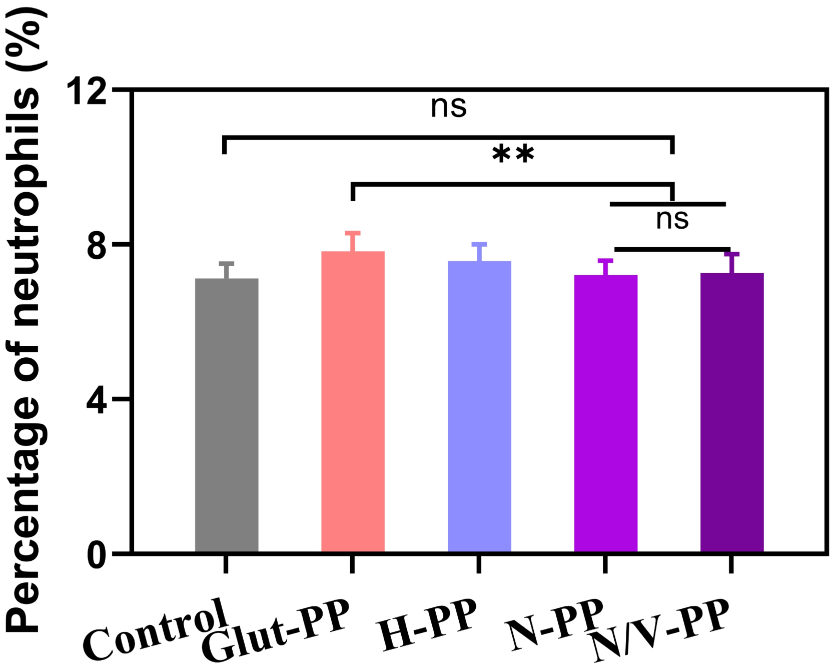

Figure S5 Percentage of neutrophils.

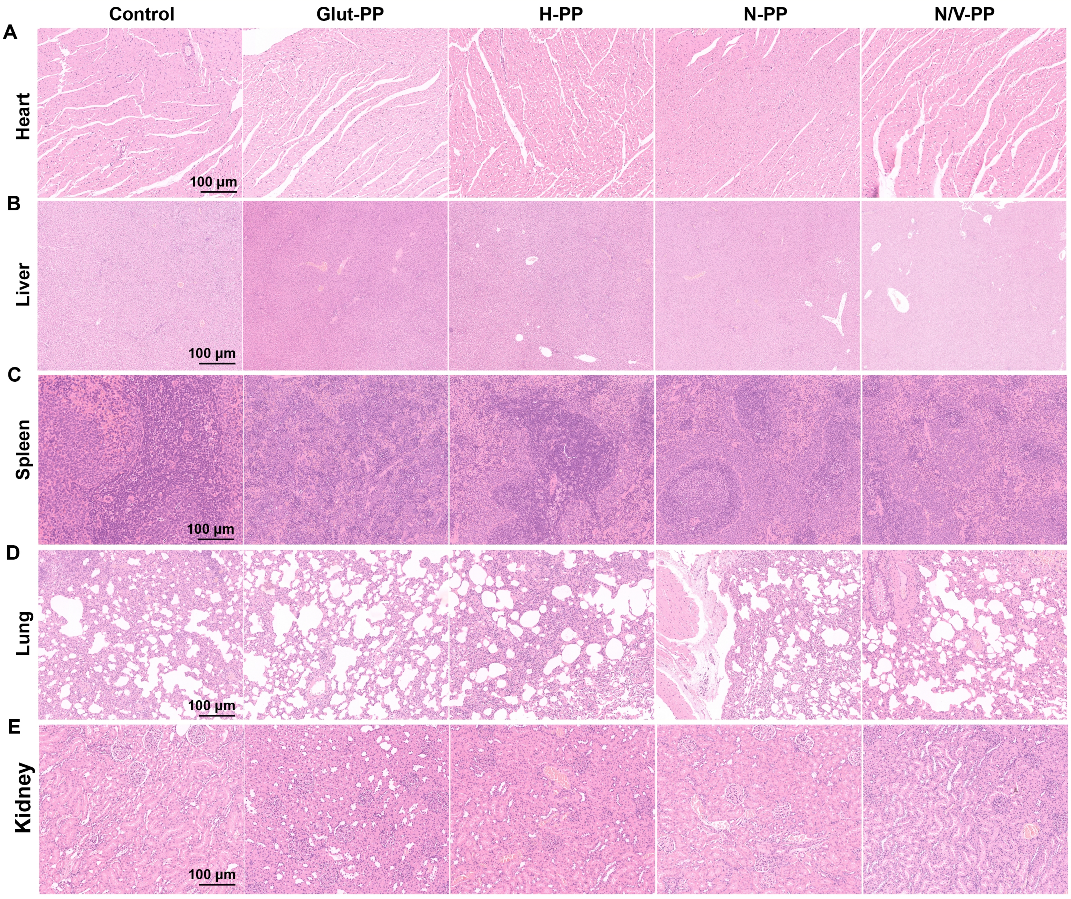

Figure S6 Histological analysis of (A) heart, (B) liver, (C) spleen, (D) lungs and (E) kidneys by H&E staining.
